# Epigenetic clocks derived from western samples differentially reflect Taiwanese health outcomes

**DOI:** 10.3389/fgene.2023.1089819

**Published:** 2023-02-06

**Authors:** Wan-Yu Lin

**Affiliations:** ^1^ Institute of Epidemiology and Preventive Medicine, College of Public Health, National Taiwan University, Taipei, Taiwan; ^2^ Master of Public Health Degree Program, College of Public Health, National Taiwan University, Taipei, Taiwan; ^3^ Department of Public Health, College of Public Health, National Taiwan University, Taipei, Taiwan

**Keywords:** DNA methylation, epigenetic age acceleration, epigenetic clock, longevity, biological age

## Abstract

**Introduction:** Several epigenetic clocks have been developed, with five measures of epigenetic age acceleration (EAA) especially receiving extensive investigations: HannumEAA, IEAA, PhenoEAA, GrimEAA, and DunedinPACE. These epigenetic clocks were mainly developed by individuals of European or Hispanic ancestry. It remains unclear whether they can reflect disease morbidity and physiological conditions in Asian populations.

**Methods:** I here investigated five measures of EAA of 2,474 Taiwan Biobank participants with DNA methylation data. Using logistic regressions, I sequentially regressed various health outcomes on each of the five measures of EAA while adjusting for chronological age, sex, body mass index, the number of smoking pack-years, drinking status, regular exercise, educational attainment, and six cell-type proportions.

**Results:** Except for IEAA, all measures of EAA reflected the obesity of Taiwanese (*p* < 4.0E-4). Diabetes was reflected by DunedinPACE (*p* = 5.4E-6) and GrimEAA (*p* = 5.8E-5). Moreover, DunedinPACE was associated with dyslipidemia, including hypertriglyceridemia (*p* = 1.1E-5), low high-density lipoprotein cholesterol (HDL-C) (*p* = 4.0E-5), and high triglyceride to HDL-C ratio (*p* = 1.6E-7).

**Discussion:** This is one of the first studies to show that epigenetic clocks (developed by individuals of European or Hispanic ancestry) can reflect Taiwanese physiological conditions. DunedinPACE was associated with more Taiwanese health outcomes than the other four measures of EAA.

## Introduction

Given the advancement of epigenetics, several epigenetic clocks have been developed to estimate human biological age, with five especially receiving much attention (Hannum et al., 2013; Horvath 2013; Levine et al., 2018; Lu et al., 2019; Belsky et al., 2022). Epigenetic age acceleration (EAA), usually obtained from the residuals of regressing epigenetic age on chronological age, measures whether people are aging faster than their chronological age (Jain et al., 2022; Lo and Lin 2022). EAA can provide important insights into human health (Horvath et al., 2016).

The earliest among the five is Hannum’s clock (Hannum et al., 2013), in which the aging model was built with more than 450,000 cytosine-phosphate-guanine (CpG) sites from the whole blood of 426 Caucasian and 230 Hispanic individuals. With the elastic net regression (Zou and Hastie 2005), Hannum *et al.* selected 71 from the ∼450,000 CpGs to predict chronological age (Hannum et al., 2013).

By using 82 data sets (*n* = 7,844), Horvath further developed a multi-tissue predictor of age, allowing to estimate the DNA methylation (DNAm) age of many tissues and cell types (Horvath 2013). A total of 353 CpGs were selected by regressing a calibrated version of chronological age on 21,369 CpGs with the elastic net regression (Zou and Hastie 2005). A weighted sum of these CpGs formed Horvath’s clock (Horvath 2013). The abovementioned Hannum *et al.*‘s clock (Hannum et al., 2013) and Horvath’s clock (Horvath 2013) are called “the first-generation epigenetic clocks”, as they are used to estimate chronological age rather than biological age. Predicting chronological age can be an important topic in forensic medicine when an individual’s chronological age is unknown.

In 2018, Levine *et al.* proposed a novel two-step approach and found hundreds of CpGs to form the so-called “PhenoAge” epigenetic clock (Levine et al., 2018). By analyzing the data of the third National Health and Nutrition Examination Survey (NHANES III, including Americans of European ancestry or African ancestry and Mexican-American persons), they first built a “phenotypic age” model by incorporating nine clinical biomarkers and chronological age. In the second step, the elastic net regression (Zou and Hastie 2005) was used to select 513 from among 20,169 CpGs as predictors of the ‘phenotypic age’. A linear combination of these CpGs formed the third epigenetic clock, “PhenoAge”.

In 2019, Lu *et al.* used a two-stage approach to build the fourth epigenetic clock, “GrimAge” (Lu et al., 2019). They first correlated the levels of 88 plasma proteins (measured from immunoassays) and self-reported smoking pack-years with DNAm values from the Framingham Heart Study data (Dawber et al., 1951). Through this step, plasma proteins and self-reported smoking pack-years were found to be estimated by some CpGs. The linear combinations of these selected CpGs are called “DNAm-based surrogate markers of plasma proteins and smoking pack-years”. In the second stage, with the elastic net Cox regression, Lu *et al.* regressed time-to-death on DNAm-based surrogate markers of plasma proteins and smoking pack-years while adjusting for sex and chronological age. DNAm-based biomarkers for smoking pack-years and seven plasma proteins were selected to reflect time-to-death. The union of these eight sets of DNAm markers, a total of 1,030 CpGs, formed the epigenetic clock, “GrimAge”.

Recently, Belsky *et al.* proposed DunedinPACE to estimate the pace of aging (Belsky et al., 2022). DunedinPACE was built by analyzing the longitudinal data from 1,037 babies born in Dunedin, New Zealand, during 1972–1973 (Belsky et al., 2022). The majority (93%) of this cohort was of European descent (Poulton et al., 2015). Based on the elastic net regression (Zou and Hastie 2005), Belsky *et al.* linked the aging pace across 2 decades of these individuals to their DNAm probes with good test-retest reliability (i.e., probes with slight variation across technical replicates). DunedinPACE was further evaluated in five additional datasets, in which most individuals were also of European descent.

PhenoAge, GrimAge, and DunedinPACE can be regarded as “the second-generation epigenetic clocks” because they are linked to “phenotypic age” (a combination of various phenotypes such as creatinine, albumin, etc.) (Levine et al., 2018), time-to-death due to all-cause mortality (Lu et al., 2019), and declines in organ-system integrity (Belsky et al., 2022), respectively. Therefore, these three epigenetic clocks can better reflect physiological conditions than the first-generation clocks.

Among the five abovementioned epigenetic clocks, four earlier clocks have been compared by some studies (Lu et al., 2019; Maddock et al., 2020; McCrory et al., 2021). For example, Lu *et al.* (Lu et al., 2019) applied the four earlier epigenetic clocks to an extensive validation data set (6,935 individuals) comprising three ethnic groups (50% European ancestry, 40% African Americans, and 10% Hispanic ancestry). They showed that GrimAge outperformed the other three clocks regarding its ability to predict time to death, time to coronary heart disease, time to cancer, etc (Lu et al., 2019).

By investigating 709 Scottish individuals, Hillary *et al.* have shown that GrimAge is associated with various measures of brain health and can help predict cognitive functions (Hillary et al., 2021). Through analyzing data from three British cohorts, Maddock *et al.* found that PhenoAge and GrimAge were significantly associated with three of the five measures of cognitive performance or functional ability (Maddock et al., 2020). McCrory *et al.* further demonstrated that GrimAge was superior to the other three clocks in predicting age-related phenotypes and all-cause mortality (McCrory et al., 2021).

All five abovementioned epigenetic clocks were mainly developed by European, African, or Hispanic individuals (Hannum et al., 2013; Horvath 2013; Levine et al., 2018; Lu et al., 2019; Belsky et al., 2022). However, notable differences in DNAm age may exist between several ethnic groups. As shown by Levine *et al.*, there are significant differences in PhenoAge between ethnic groups in the Women’s Health Initiative data set (Levine et al., 2018). On average, individuals of African ancestry had the highest PhenoAge, whereas individuals of European ancestry generally had the lowest PhenoAge. Hispanics were between them but close to people of African ancestry.

Despite these abundant studies in DNAm age, very few have been conducted using data from Asian populations. Whether these five epigenetic clocks can reflect Asians’ disease morbidity or physiological conditions remains unknown. In this work, I calculated five measures of EAA using the DNAm data of 2,474 Taiwan biobank (TWB) participants. As DNAm age has received extensive attention, exploring the EAA measure that can most effectively reflect Taiwanese health outcomes will be essential.

## Methods

### Taiwan biobank

TWB was approved by the Institutional Review Board on Biomedical Science Research/IRB-BM, Academia Sinica, and also by the Ethics and Governance Council of Taiwan Biobank, Taiwan. The current study further received approval from the Research Ethics Committee of National Taiwan University Hospital (NTUH-REC no. 201805050RINB). Written informed consent was obtained from each participant following institutional requirements and the principles of the Declaration of Helsinki.

Since October 2012, TWB has recruited 160,808 community-based volunteers aged 30–70 years. The majority of TWB individuals were of Han Chinese ancestry (Chen et al., 2016). After a fast for at least 6 h, each TWB participant provided blood and urine samples and took physical examinations. Lifestyle information was further collected through a face-to-face interview with TWB researchers. During 2016–2021, 2,474 TWB participants were randomly selected for DNAm analysis. The Illumina Infinium MethylationEPIC BeadChip (Illumina, Inc., San Diego, CA) covering ∼860,000 CpG sites was used to quantify their blood DNAm values.

### Calculation of five epigenetic clocks

DNAm intensity data were normalized by the normal-exponential out-of-band (noob) approach (Triche et al., 2013) with the *preprocessNoob* function in the R package minfi v1.36 (Aryee et al., 2014). The TWB DNAm data were then uploaded to the online DNAm Age Calculator developed by Horvath’s laboratory, https://dnamage.genetics.ucla.edu/new. The Illumina Infinium MethylationEPIC BeadChip included 91.5% of the 30,084 sites (i.e., 27,526 CpGs) listed in the annotation file “datMiniAnnotation3. csv” under Horvath’s “Advanced Analysis”. The average detection *p-*value across the 27,526 CpGs was used to evaluate the quality of DNAm quantification for each sample. Because all samples’ average detection *p-*values were much smaller than the suggested cutoff 0.01 (Maksimovic et al., 2016), the quality of the DNAm data was regarded as satisfactory.

The measures of EAA based on four epigenetic clocks were extracted from the columns “AgeAccelerationResidualHannum” (Hannum *et al.*‘s clock (Hannum et al., 2013)), “IEAA” (Horvath’s clock (Horvath 2013)), “AgeAccelPheno” (Levine *et al.*‘s clock (Levine et al., 2018)), and “AgeAccelGrim” (Lu *et al.*‘s clock (Lu et al., 2019)) of the output from Horvath’s DNAm age calculator, respectively. Furthermore, DunedinPACE (Belsky et al., 2022) was calculated using the R package DunedinPACE, available from GitHub at https://github.com/danbelsky/DunedinPACE.

### Health outcomes

I here investigated the associations of EAA measures with metabolic conditions, cardiovascular health, physical activity, and lung function. Metabolic conditions included obesity (body mass index [BMI] > 27 kg/m^2^), adiposity (male body fat percentage [BFP] > 25% or female BFP >30%), abdominal obesity (male waist circumference [WC] > 90 cm or female WC > 80 cm), diabetes (with physician-diagnosed diabetes, or fasting glucose >126 mg/dL or glycated hemoglobin [HbA1c] > 6.5% based on TWB test results), hypertension (with physician-diagnosed hypertension, or diastolic blood pressure [DBP] > 80 mmHg or systolic blood pressure [SBP] > 130 mmHg based on TWB test results), hypertriglyceridemia (triglyceride [TG] > 150 mg/dL), low-density lipoprotein cholesterol [LDL-C] > 130 mg/dL, low high-density lipoprotein cholesterol (HDL-C) (male HDL-C < 40 mg/dL or female HDL-C < 50 mg/dL), high TG/HDL-C ratio (male TG/HDL-C > 3.75 or female TG/HDL-C > 3).

The cutoff values for BMI, BFP, WC, TG, LDL-C, and HDL-C were based on Taiwan’s Ministry of Health and Welfare (MoHW) recommendations. The definition of obesity (BMI ≥27 kg/m^2^) is more suitable for Asians, although it is more stringent than the criterion defined by the World Health Organization (BMI ≥30 kg/m^2^). According to Taiwan’s MoHW, dyslipidemia refers to unhealthy levels for at least one type of lipid, including TG > 150 mg/dL, LDL-C level >130 mg/dL, and male HDL-C level <40 mg/dL or female HDL-C level <50 mg/dL. Moreover, TG to HDL-C (the TG/HDL-C ratio) is a useful predictor for identifying cardiometabolic risk (Murguia-Romero et al., 2013). Therefore, the TG/HDL-C ratio cutoff is defined as 3.75 (= 150/40) for males or 3 (= 150/50) for females. In addition to these dichotomous traits, I also analyzed 11 health outcome measurements without dichotomization.

BFP was measured by bioelectrical impedance analysis using a TANITA Body composition analyzer BC-420MA (Tokyo, Japan). After a fast for at least 6 h, serum HbA1c and glucose levels were measured with the Trinity Biotech Premier Hb9210 analyzer (Bray, Ireland/Kansas City, MO) and the Hitachi LST008 analyzer (Hitachi High-Technologies, Tokyo, Japan), respectively. To obtain more reliable DBP and SBP, I used the average of two measured blood pressure levels (a 5-min rest interval between the two measurements).

Cardiovascular health outcomes included coronary artery disease (CAD) and cardiovascular diseases (CVDs). CVDs indicated valvular heart disease, CAD, arrhythmia, cardiomyopathy, congenital heart disease, apoplexy, and other diseases involving blood vessels or the heart.

I also assessed the relationship between these 5 measures of EAA and regular exercise (yes vs. no). Regular exercise was defined as performing 30 min of “exercise” three times a week. “Exercise” included leisure-time activities such as walking, brisk walking, jogging, swimming, dancing, weight training, badminton, table tennis, mountain climbing, etc. A total of 1,092 out of the 2,474 individuals (44%) developed the habit of regular exercise. Subjects with regular exercise were further surveyed on what kind of exercise they usually performed within the last 3 months. Walking is a less physically demanding activity and may be chosen by subjects with limited exercise capacity. I here investigated the associations of these 5 measures of EAA with “choosing walking as the regular exercise”. Finally, lung function was assessed by forced vital capacity (FVC).

### Statistical analysis

The logistic regression was performed for each dichotomous health outcome. Each outcome (yes vs. no) was regressed on the *z*-score transformation of EAA while adjusting for sex, chronological age (in years), BMI, the number of smoking pack-years, drinking status (yes vs. no), regular exercise (yes vs. no), educational attainment (an integer ranging from 1 to 7), and six cell-type proportions (B lymphocytes, CD4^+^ T cells, CD8^+^ T cells, monocytes, natural killer cells, and neutrophils). These immune cell proportions were estimated by the Houseman deconvolution method (Houseman et al., 2012), because this is the optimal among reference-based deconvolution methods (Kaushal et al., 2017; Titus et al., 2017).

Non-etheless, BMI was not adjusted when the health outcome (the response variable) was obesity, adiposity, or abdominal obesity. Likewise, regular exercise was not adjusted when the response variable was regular exercise (itself) or “choosing walking as the regular exercise” (because all the 1,092 individuals performed regular exercise in this analysis).

FVC and 11 metabolic traits (without dichotomization) were continuous. I first calculated the residuals of regressing the continuous traits on the 13 covariates mentioned above, becoming “covariates-adjusted traits”. Using Spearman’s rank correlation coefficient, I then assessed the association between each covariates-adjusted trait and each EAA measure. The *p*-value for testing no correlation between covariates-adjusted trait and EAA was also presented. Given Spearman’s rank correlation coefficients, the magnitudes of associations with EAA measures can be compared across different continuous traits.

Results with *p* < 4.0E-4 $=0.05/\left(25\times 5\right)$ were regarded as statistically significant, according to the Bonferroni correction for five measures of EAA and 25 health outcomes (14 health outcomes and 11 continuous metabolic traits). The *p*-values for testing associations of EAA measures with health outcomes were based on logistic regression and Spearman’s rank correlation coefficient for dichotomous and continuous traits, respectively.

## Results

### Basic characteristics of the 2,474 TWB participants

The basic characteristics of the 2,474 TWB participants are shown in Table 1. Among the 2,474 individuals, 1,243 (50.2%) were males while 1,231 (49.8%) were females. The chronological age ranged from 30 to 70 years, with averages of 50.3 (standard deviation, s.d. = 11.3) and 49.3 (s.d. = 10.8) years for males and females, respectively. All epigenetic clocks except for PhenoEAA showed that males had a significantly faster aging rate than females (*p*<=2.0E-11). For example, the result of DunedinPACE showed an average of 1.005 (s.d. = 0.108) biological years per chronological year for males and an average of 0.976 (s.d. = 0.104) biological years per chronological year for females.

**TABLE 1:** Basic characteristics of the 2,474 TWB participants.

	Males	Females	*p*-value^a^
Total	1,243 (50.2%)	1,231 (49.8%)	
Chronological age (years)	50.3 ± 11.3	49.3 ± 10.8	0.0246
HannumEAA (years)	0.68 ± 3.73	−0.69 ± 3.70	8.5E-20
IEAA (years)	0.66 ± 3.68	−0.67 ± 3.63	2.6E-19
PhenoEAA (years)	0.05 ± 4.69	−0.05 ± 5.14	0.5854
GrimEAA (years)	1.43 ± 3.62	−1.44 ± 2.71	5.0E-100
DunedinPACE	1.005 ± 0.108	0.976 ± 0.104	2.0E-11
Drinking^b^	147 (11.8%)	24 (1.9%)	7.7E-22
Smoking^c^	235 (18.9%)	48 (3.9%)	2.0E-31
The number of pack-years for smokers	22.3 ±20 .8	10.8 ± 10.3	6.2E-8
Regular exercise^d^	595 (47.9%)	497 (40.4%)	2.0E-4
Educational attainment^e^	5.8 ± 0.9	5.4 ± 0.9	1.6E-22
BMI (kg/m^2^)	25.2 ± 3.4	23.5 ± 3.7	2.3E-32
Obesity (BMI >27 kg/m^2^)	333 (26.8%)	196 (15.9%)	6.0E-11
Body fat percentage (%)	22.9 ± 5.4	31.8 ± 6.5	4.5E-228
Adiposity^f^	381 (30.7%)	687 (55.8%)	2.4E-36
Waist circumference (cm)	87.9 ± 9.3	80.5 ± 9.8	6.8E-76
Abdominal obesity^g^	442 (35.6%)	561 (45.6%)	4.9E-7
Diabetes^h^	122 (9.8%)	67 (5.4%)	5.9E-5
Hypertension^i^	586 (47.1%)	300 (24.4%)	5.5E-32
Coronary artery disease	35 (2.8%)	7 (0.57%)	3.0E-5
Cardiovascular diseases^j^	140 (11.3%)	136 (11.0%)	0.9155
Hypertriglyceridemia^k^	319 (25.7%)	161 (13.1%)	3.7E-15
High LDL-C^l^	469 (37.7%)	417 (33.9%)	0.0502
Low HDL-C^m^	274 (22.0%)	318 (25.8%)	0.0306
High TG/HDL-C ratio^n^	300 (24.1%)	181 (14.7%)	4.2E-9
“walking” as the regular exercise	183 (14.7%)	183 (14.9%)	0.9650
Forced vital capacity (FVC, in mL)	3623 ± 687 (811 males measured FVC)	2537 ± 613 (765 females measured FVC)	3.8E-183

Data are presented in *n* (%) or mean ± standard deviation.

^a^ *p*-value of testing the mean difference between males and females, based on the two-sample *t*-test (for continuous variables) or proportion test (for dichotomous variables).

^b^ Drinking was defined as a person having a weekly intake of more than 150 mL of alcoholic beverages for at least 6 months and having not stopped drinking at the time he/she participated in TWB.

^c^ Smoking was defined as a person who had smoked cigarettes for at least 6 months and had not quit smoking at the time he/she participated in TWB.

d Regular exercise was defined as performing 30 min of “exercise” three times a week. “Exercise” included leisure-time activities such as walking, jogging, swimming, cycling, dancing, weight training, mountain climbing, etc.

e Educational attainment ranged from 1 to 7: 1 “illiterate”, 2 “no formal education but literate”, 3 “primary school graduate”, 4 “junior high school graduate”, 5 “senior high school graduate”, 6 “college graduate”, and 7 “Master’s or higher degree”.

^f^ Adiposity: Male BFP >25% or female BFP >30%.

^g^ Abdominal obesity: Male WC > 90 cm or female WC > 80 cm.

^h^ Individuals with diabetes included those with physician-diagnosed diabetes, or subjects having fasting glucose >126 mg/dL or HbA1c > 6.5% (48 mmol/mol) based on TWB test results.

i Individuals with hypertension included those with physician-diagnosed hypertension, or subjects having DBP >80 mmHg or SBP >130 mmHg based on TWB test results.

^j^ Cardiovascular diseases (CVDs) included the diagnosis of valvular heart disease, coronary artery disease, arrhythmia, cardiomyopathy, congenital heart disease, and apoplexy or any other diseases involving blood vessels or heart.

^k^ Hypertriglyceridemia: TG > 150 mg/dL.

^l^ High LDL-C: LDL-C > 130 mg/dL.

^m^ Low HDL-C: Male HDL-C < 40 mg/dL or female HDL-C < 50 mg/dL.

^n^ High TG/HDL-C ratio: male TG/HDL-C > 3.75 (= 150/40) or female TG/HDL-C > 3 (= 150/50).

**FIGURE 1:**
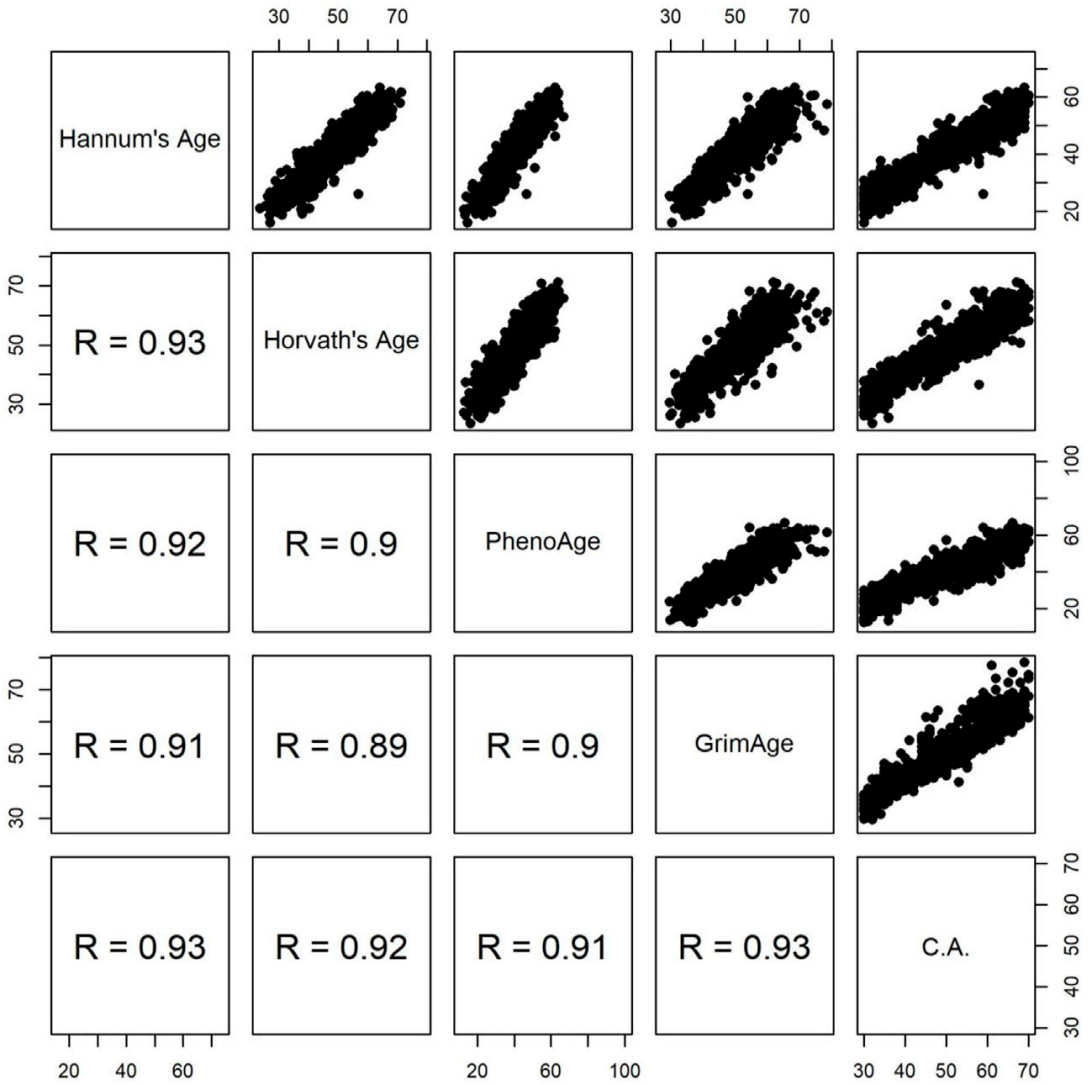
Pairwise scatter plots of four measures of DNAm age and chronological age (C.A). R represents Spearman’s rank correlation coefficient.

**FIGURE 2:**
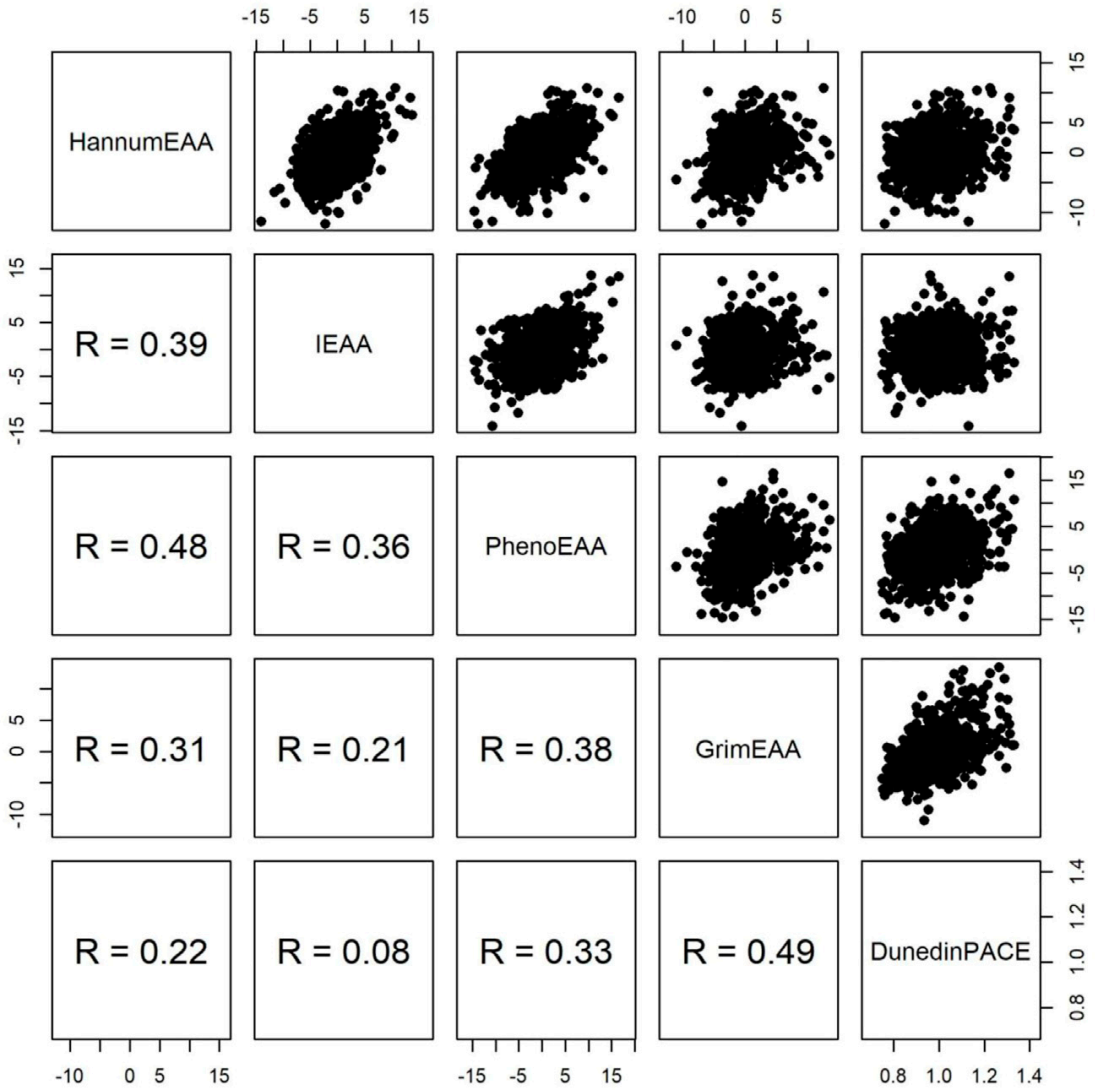
Pairwise scatter plots of four measures of epigenetic age acceleration and DunedinPACE. R represents Spearman’s rank correlation coefficient.

While DunedinPACE measures the pace of aging (Belsky et al., 2022), the other four clocks estimate biological age (Hannum et al., 2013; Horvath 2013; Levine et al., 2018; Lu et al., 2019). Figure 1 shows the pairwise scatter plots of the four measures of DNAm age and chronological age. All four measures of DNAm age are highly correlated with chronological age (Spearman’s rank correlation coefficient ≥ 0.90, as shown by the bottom row of Figure 1) (Spearman’s rank correlation coefficient was used throughout this study for its robustness to outliers). Four measures of EAA were obtained by residuals of regressing the respective DNAm age on the chronological age. In this way, EAA can be robust to different measurement platforms and normalization methods (McEwen et al., 2018). Figure 2 shows the pairwise scatter plots of four measures of EAA and DunedinPACE. The largest Spearman’s rank correlation coefficient is 0.49 between the latest two clocks, i.e., GrimEAA (Lu et al., 2019) and DunedinPACE (Belsky et al., 2022).

Table 1 shows that sex difference was significant in most health outcomes (*p* < 0.05), except for CVDs (*p* = 0.9155), “high LDL-C” (*p* = 0.0502) and “choosing walking as the regular exercise” (*p* = 0.9650). Therefore, sex would be adjusted in all (logistic) regression models.

FVC was used to evaluate individuals’ lung function. It measured the total exhaled volume of air after a maximal inspiration. Approximately 64% of the 2,474 TWB participants underwent this respiratory examination. The mean FVC was 3,623 (s.d. = 687) mL for 811 males and 2,537 (s.d. = 613) mL for 765 females.

### Epigenetic age acceleration and health outcomes

Before assessing the associations of EAA with health outcomes, I removed extreme outliers of EAA if they were smaller than $Q_{1}-3\times \left(Q_{3}-Q_{1}\right)$ or larger than $Q_{3}+3\times \left(Q_{3}-Q_{1}\right)$ , where $Q_{1}$ and $Q_{3}$ are the 25th and 75th percentiles of an EAA, respectively. Through this step, a total of 7, 1, 2, 5, and 1 extreme outliers were excluded for HannumEAA, IEAA, PhenoEAA, GrimEAA, and DunedinPACE analyses, respectively.

**FIGURE 3:**
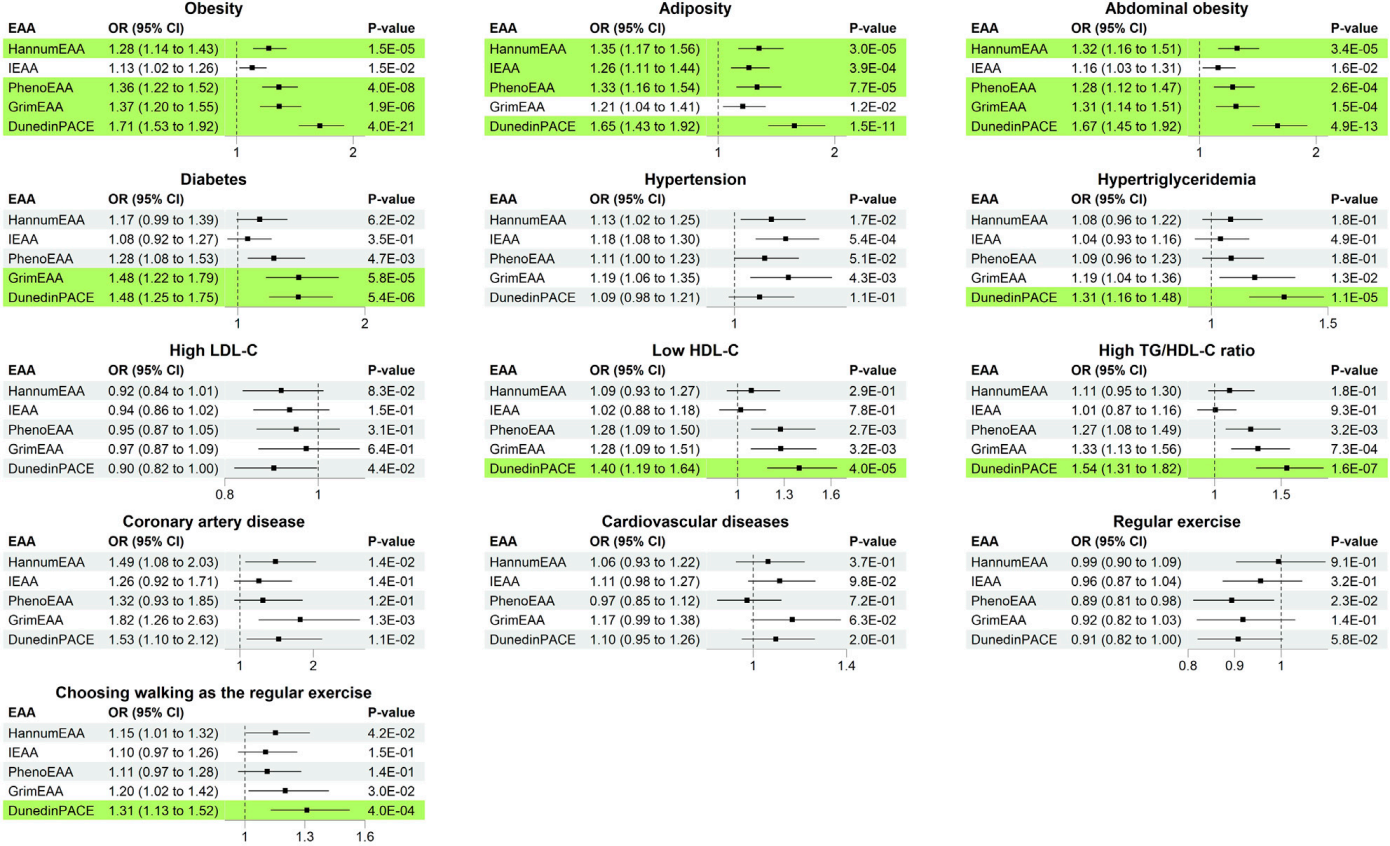
Odds ratio (with 95% C.I. and *p*-values) of increasing one standard deviation of epigenetic age acceleration. Significant results with *p* < 0.05/(25 × 5) = 4.0E-4 were marked in green background.

After removing the extreme outliers from respective analyses, I investigated the associations of the five measures of EAA sequentially with the 14 health outcomes (one outcome at a time). Figure 3 presents the analysis results of 13 dichotomous outcomes (FVC was continuous and was summarized in Table 2). DunedinPACE was associated with 8/14 health outcomes. For example, an s.d. increase in DunedinPACE was associated with an OR of 1.48 to develop diabetes (95% C.I. = 1.25–1.75, *p* = 5.4E-6), an OR of 1.67 to develop abdominal obesity (95% C.I. = 1.45–1.92, *p* = 4.9E-13), an OR of 1.31 to develop hypertriglyceridemia (95% C.I. = 1.16–1.48, *p* = 1.1E-5), etc.

**TABLE 2:** Spearman’s rank correlation coefficients (with 95% C.I. and *p*-values) between epigenetic age acceleration and continuous health outcomes. Significant results with *p* < 0.05/(25 × 5) = 4.0E-4 were highlighted in bold font.

12 continuous health outcomes	HannumEAA (s.d. = 3.63 years)				IEAA (s.d. = 3.69 years)				PhenoEAA (s.d. = 4.79 years)				GrimEAA (s.d. = 3.43 years)				DunedinPACE (s.d. = 0.1064)			
	Spearman’s correlation	95% CI		*p*-value	Spearman’s correlation	95% CI		*p*-value	Spearman’s correlation	95% CI		*p*-value	Spearman’s correlation	95% CI		*p*-value	Spearman’s correlation	95% CI		*p*-value
**Body mass index**	**0.089**	**0.050**	**0.128**	**9.8E-06**	0.058	0.019	0.098	3.7E-03	**0.115**	**0.076**	**0.154**	**1.1E-08**	**0.115**	**0.076**	**0.154**	**1.0E-08**	**0.238**	**0.200**	**0.275**	**4.1E-33**
**Body fat percentage**	**0.084**	**0.044**	**0.124**	**3.7E-05**	0.046	0.006	0.086	2.3E-02	**0.101**	**0.061**	**0.140**	**7.9E-07**	**0.104**	**0.064**	**0.144**	**3.6E-07**	**0.217**	**0.178**	**0.255**	**7.8E-27**
**Waist circumference**	**0.084**	**0.044**	**0.123**	**3.2E-05**	0.048	0.009	0.087	1.7E-02	**0.106**	**0.066**	**0.144**	**1.5E-07**	**0.110**	**0.071**	**0.149**	**4.1E-08**	**0.209**	**0.171**	**0.247**	**8.4E-26**
**Fasting glucose**	−0.011	−0.051	0.028	5.8E-01	−0.025	−0.064	0.015	2.2E-01	−0.029	−0.069	0.010	1.5E-01	−0.049	−0.088	−0.010	1.5E-02	**−0.109**	**−0.148**	**−0.070**	**5.3E-08**
**HbA1c**	0.014	−0.026	0.053	4.9E-01	−0.010	−0.050	0.029	6.2E-01	0.014	−0.026	0.053	4.9E-01	0.003	−0.037	0.042	8.9E-01	−0.019	−0.059	0.020	3.4E-01
**Diastolic blood pressure**	0.026	−0.013	0.066	1.9E-01	0.056	0.017	0.095	5.4E-03	0.025	−0.015	0.064	2.2E-01	0.007	−0.032	0.047	7.2E-01	0.017	−0.022	0.057	3.9E-01
**Systolic blood pressure**	0.026	−0.013	0.066	1.9E-01	0.031	−0.009	0.070	1.3E-01	0.011	−0.029	0.050	5.9E-01	0.017	−0.023	0.056	4.1E-01	0.023	−0.017	0.062	2.6E-01
**Triglyceride**	0.020	−0.020	0.059	3.3E-01	−0.008	−0.048	0.031	6.8E-01	0.027	−0.013	0.066	1.9E-01	−0.014	−0.053	0.026	5.0E-01	0.026	−0.013	0.066	1.9E-01
**LDL-C**	−0.016	−0.055	0.024	4.3E-01	−0.016	−0.055	0.024	4.4E-01	−0.023	−0.062	0.017	2.6E-01	−0.009	−0.049	0.030	6.5E-01	−0.039	−0.079	0.000	5.1E-02
**HDL-C**	−0.043	−0.082	−0.003	3.5E-02	−0.013	−0.052	0.027	5.3E-01	−0.039	−0.078	0.001	5.4E-02	−0.068	−0.107	−0.029	7.0E-04	**−0.131**	**−0.170**	**−0.092**	**6.5E-11**
**TG/HDL-C ratio**	0.029	−0.010	0.069	1.5E-01	−0.009	−0.048	0.031	6.6E-01	0.035	−0.005	0.074	8.5E-02	−0.022	−0.061	0.018	2.8E-01	0.029	−0.011	0.068	1.6E-01
**Forced vital capacity**	−0.020	−0.069	0.030	4.3E-01	−0.003	−0.052	0.046	9.1E-01	0.004	−0.045	0.054	8.6E-01	−0.004	−0.054	0.045	8.7E-01	−0.003	−0.053	0.046	8.9E-01

GrimEAA was associated with 3/14 health outcomes, including diabetes (*p* = 5.8E-5), abdominal obesity (*p* = 1.5E-4), and obesity (*p* = 1.9E-6). An s.d. increase in GrimEAA (3.43 years) was associated with an OR of 1.48 to develop diabetes (95% C.I. = 1.22–1.79), an OR of 1.31 to develop abdominal obesity (95% C.I. = 1.14–1.51), and an OR of 1.37 to develop obesity (95% C.I. = 1.20–1.55).

PhenoEAA and HannumEAA were associated with 3/14 health outcomes, including obesity, adiposity, and abdominal obesity (*p* < 4.0E-4). An s.d. increase in PhenoEAA (4.79 years) or HannumEAA (3.63 years) was associated with a higher risk of developing obesity-related outcomes.

I further calculated Spearman’s rank correlation coefficients between the five measures of EAA and 11 adjusted metabolic traits (adjusted for covariates, explained in Materials and Methods). Through Spearman’s rank correlation coefficients, I may compare the magnitudes of associations with EAA measures across different continuous traits. The results are presented in Table 2. Except for IEAA, all measures of EAA were significantly positively correlated with three obesity traits (*p* < 4.0E-4), i.e., BMI, BFP, and WC. DunedinPACE provided the most considerable magnitude of correlations with the three obesity traits (Spearman’s rank correlation coefficients >0.2, Table 2). Furthermore, DunedinPACE was significantly negatively correlated with fasting glucose (*p* = 5.3E-8) and HDL-C (*p* = 6.5E-11).

### Epigenetic age acceleration and regular exercise

A total of 595 males (47.9%) and 497 females (40.4%) had developed the habit of regular exercise. None of the five EAA measures were associated with performing regular exercise (*p* > 4.0E-4, Figure 3). Subjects with regular exercise also provided the activity they usually engaged in within the last 3 months, including walking, brisk walking, jogging, swimming, dancing, weight training, badminton, table tennis, mountain climbing, etc. Walking is a less physically demanding exercise and is more likely to be chosen by subjects with limited exercise capacity.

Among the five measures of EAA, DunedinPACE was significantly associated with “choosing walking as the regular exercise” (*p* = 4.0E-4). An s.d. increase in DunedinPACE was associated with an OR of 1.31 to choose walking as the regular exercise (95% C.I. = 1.13–1.52). Among the individuals with regular exercise, people with larger levels of DunedinPACE were more likely to choose walking as the regular exercise (implying that they may have a limited exercise capacity). The other four measures of EAA were also in the same direction regarding their associations with “choosing walking as the regular exercise” (OR > 1.0, Figure 3). However, they were not as significant as DunedinPACE.

For future meta-analyses, I also listed the results from minimally adjusted models in the Supplementary Materials, i.e., models adjusted only for age and sex. Results of minimally adjusted models also showed that DunedinPACE is associated with more Taiwanese health outcomes than the other four measures of EAA.

### Epigenetic age acceleration and lifestyle factors

To explore how such environmental and lifestyle factors may similarly or differently impact epigenetic models of aging in Asian populations, I regressed each EAA on the seven main covariates while adjusting for the six cell-type proportions. Covariates with *p* < 0.05/(5 × 7) = 1.4E-3 were regarded as significant, according to the Bonferroni correction considering five measures of EAA and seven main covariates.

Table 3 shows that alcohol drinking was not significantly associated with any EAA (*p* > 1.4E-3). In my data, alcohol drinking was defined as a person having a weekly intake of more than 150 mL of alcoholic beverages for at least 6 months and having not stopped drinking at the time he/she participated in TWB. People with moderate and heavy levels of alcohol consumption were all categorized as “alcohol drinking” in the TWB data. Therefore, I may not detect the protective effect against aging for moderate alcohol consumption.

**TABLE 3:** Results of regressing each EAA on the seven main covariates (six cell-type proportions were also adjusted in the models).

	HannumEAA (in years)		IEAA (in years)		PhenoEAA (in years)		GrimEAA (in years)		DunedinPACE	
	Effect estimate	*p*-value	Effect estimate	*p*-value	Effect estimate	*p*-value	Effect estimate	*p*-value	Effect estimate	*p*-value
Chronological age (in years, continuous variable)	**−0.0349**	**6.6E-07**	0.0007	9.3E-01	−0.0205	2.6E-02	**−0.0199**	**3.6E-04**	**0.0022**	**2.2E-27**
Sex (1: male vs. 0: female)	0.3093	1.2E-01	**1.0850**	**8.7E-07**	**−1.2921**	**1.1E-06**	**1.6890**	**1.3E-25**	0.0089	1.1E-01
BMI (in kg/m^2^, continuous variable)	**0.0947**	**5.2E-07**	0.0485	1.9E-02	**0.1667**	**2.1E-11**	**0.0819**	**4.8E-08**	**0.0070**	**3.5E-38**
The number of smoking pack-years (continuous)	**0.0251**	**8.1E-04**	0.0003	9.7E-01	**0.0360**	**2.8E-04**	**0.1535**	**1.3E-124**	**0.0023**	**1.6E-27**
Drinking status (1: yes vs. 0: no)	0.5196	5.4E-02	0.2879	3.3E-01	0.8320	1.9E-02	0.4617	3.2E-02	0.0047	5.4E-01
Regular exercise (1: yes vs. 0: no)	−0.0200	8.9E-01	−0.1783	2.6E-01	−0.4483	1.9E-02	−0.1664	1.5E-01	−0.0075	6.5E-02
Educational attainment (an integer of 1–7)	−0.1053	1.7E-01	−0.0318	7.1E-01	−0.2901	4.1E-03	**−0.2421**	**7.4E-05**	**−0.0100**	**3.9E-06**

Significant results with *p* < 0.05/(5 × 7) = 1.4E-3 were highlighted in bold font (5: Five measures of EAA; 7: Seven main covariates).

Regarding cigarette smoking, I found that the number of pack-years was significantly associated with an increase in HannumEAA (*p* = 8.1E-4), PhenoEAA (*p* = 2.8E-4), GrimEAA (*p* = 1.3E-124), and DunedinPACE (*p* = 1.6E-27). This result is similar to that observed in HannumEAA of other ancestries (Beach et al., 2015).

## Discussion

EAA has become a promising aging biomarker (Jylhava et al., 2017). However, studies to date have been focused on individuals of European, African, and Hispanic ancestries (Hannum et al., 2013; Horvath 2013; Levine et al., 2018; Lu et al., 2019; Maddock et al., 2020; Hillary et al., 2021; McCrory et al., 2021; Belsky et al., 2022). For example, by analyzing 490 participants from the Irish Longitudinal Study on Ageing (TILDA), McCrory *et al.* showed that GrimEAA and PhenoEAA were associated with 8/9 and 4/9 health outcomes, respectively (McCrory et al., 2021). A meta-analysis pooled 23 studies relevant to HannumEAA and IEAA, among which 11 studies assessed the associations of DNAm age with age-related diseases (Fransquet et al., 2019). Most individuals were of European, African, and Hispanic ancestries, while very few subjects were of Asian descent. All 11 but one of the studies found that increased HannumEAA or IEAA level was associated with elevated risks of diseases (Fransquet et al., 2019).

Despite emerging interest in linking epigenetic clocks to diseases and aging, replications to Asian populations have been sparse and rare. Due to significant differences observed in DNAm age between various ethnic groups (Levine et al., 2018), it is essential to investigate whether these epigenetic clocks can be applied to Asians.

Associations between lifestyle factors and first-generation EAA have been evaluated in individuals of European, African, and Hispanic ancestries (Beach et al., 2015; Quach et al., 2017). Moderate alcohol consumption was negatively associated with HannumEAA (anti-aging) (Beach et al., 2015; Quach et al., 2017), while low and heavy levels of alcohol consumption were positively associated with HannumEAA (pro-aging) (Beach et al., 2015). Cigarette smoking was also associated with an increase in HannumEAA (pro-aging) (Beach et al., 2015).

A limitation is that mortality outcome was not included due to a short follow-up time. Indeed, the blood samples of the 2,474 TWB participants were collected during 2012–2021, and very few mortality outcomes have been observed till 2022. Therefore, I here evaluated the performance of the five measures of EAA in explaining the physiological conditions of the Taiwanese. The five measures were sequentially used as a predictor to explain the health outcomes (response variable) while adjusting for sex, chronological age, BMI, the number of smoking pack-years, drinking status, regular exercise, educational attainment, and six cell-type proportions. All the significant results were described as associations rather than causality.

This is one of the first studies to apply the five measures of EAA to an Asian population. Results from samples of other ancestries indicated that DunedinPACE and GrimEAA outperformed the other clocks in measuring biological aging (Belsky et al., 2022). Moreover, DunedinPACE provided better prediction in disability than GrimEAA (Belsky et al., 2022). Belsky et al.'s finding is in line with my analysis result for the Taiwanese data, i.e., people with larger DunedinPACE were more likely to choose a less physically demanding activity (such as walking) as regular exercise (*p* = 4.0E-4, Figure 3). Through my investigation and analyses, DunedinPACE reflected more Taiwanese health outcomes than the other four measures of EAA.

## Data Availability

The data analyzed in this study is subject to the following licenses/restrictions: The individual-level Taiwan Biobank data supporting this study’s findings are available upon application to Taiwan Biobank (https://www.twbiobank.org.tw/new_web/). Requests to access these datasets should be directed to Taiwan Biobank (https://www.twbiobank.org.tw/new_web/).

## References

[B1] Aryee M. J. Jaffe A. E. Corrada-Bravo H. Ladd-Acosta C. Feinberg A. P. Hansen K. D. (2014). Minfi: A flexible and comprehensive bioconductor package for the analysis of Infinium DNA methylation microarrays. Bioinformatics 30, 1363–1369. 10.1093/bioinformatics/btu049 24478339 PMC4016708

[B2] Beach S. R. H. Dogan M. V. Lei M. K. Cutrona C. E. Gerrard M. Gibbons F. X. (2015). Methylomic aging as a window onto the influence of lifestyle: Tobacco and alcohol use alter the rate of biological aging. J. Am. Geriatrics Soc. 63, 2519–2525. 10.1111/jgs.13830 PMC4906951 26566992

[B3] Belsky D. W. Caspi A. Corcoran D. L. Sugden K. Poulton R. Arseneault L. (2022). DunedinPACE, a DNA methylation biomarker of the pace of aging. Elife 14, 11. Epub 2022/01/15. 10.7554/eLife.73420 PMC8853656 35029144

[B4] Chen C. H. Yang J. H. Chiang C. W. K. Hsiung C. N. Wu P. E. Chang L. C. (2016). Population structure of Han Chinese in the modern Taiwanese population based on 10,000 participants in the Taiwan Biobank project. Hum. Mol. Genet. 25, 5321–5331. 10.1093/hmg/ddw346 27798100 PMC6078601

[B5] Dawber T. R. Meadors G. F. Moore F. E. Jr (1951). Epidemiological approaches to heart disease: The Framingham study. Am. J. Public Health Nations Health 41, 279–281. Epub 1951/03/01. 10.2105/ajph.41.3.279 14819398 PMC1525365

[B6] Fransquet P. D. Wrigglesworth J. Woods R. L. Ernst M. E. Ryan J. (2019). The epigenetic clock as a predictor of disease and mortality risk: A systematic review and meta-analysis. Clin. Epigenetics 11, 62. 10.1186/s13148-019-0656-7 30975202 PMC6458841

[B7] Hannum G. Guinney J. Zhao L. Zhang L. Hughes G. Sadda S. (2013). Genome-wide methylation profiles reveal quantitative views of human aging rates. Mol. Cell 49 (49), 359–367. 10.1016/j.molcel.2012.10.016 23177740 PMC3780611

[B8] Hillary R. F. Stevenson A. J. Cox S. R. McCartney D. L. Harris S. E. Seeboth A. (2021). An epigenetic predictor of death captures multi-modal measures of brain health. Mol. Psychiatry. Aug 26, 3806–3816. Epub 2019/12/05. 10.1038/s41380-019-0616-9 PMC8550950 31796892

[B9] Horvath S. Gurven M. Levine M. E. Trumble B. C. Kaplan H. Allayee H. (2016). An epigenetic clock analysis of race/ethnicity, sex, and coronary heart disease. Genome Biol. 17, 171. 10.1186/s13059-016-1030-0 27511193 PMC4980791

[B10] Horvath S. (2013). DNA methylation age of human tissues and cell types. Genome Biol. 14, R115. 10.1186/gb-2013-14-10-r115 24138928 PMC4015143

[B11] Houseman E. A. Accomando W. P. Koestler D. C. Christensen B. C. Marsit C. J. Nelson H. H. (2012). DNA methylation arrays as surrogate measures of cell mixture distribution. Bmc Bioinforma. 8, 86. 10.1186/1471-2105-13-86 PMC3532182 22568884

[B12] Jain P. Binder A. M. Chen B. Parada H. Gallo L. C. Alcaraz J. (2022). Analysis of epigenetic age acceleration and healthy longevity among older US women. Jama Netw. Open 5, e2223285. 10.1001/jamanetworkopen.2022.23285 35895062 PMC9331104

[B13] Jylhava J. Pedersen N. L. Hagg S. (2017). Biological age predictors. EBioMedicine 21, 29–36. Epub 2017/04/12. 10.1016/j.ebiom.2017.03.046 28396265 PMC5514388

[B14] Kaushal A. Zhang H. Karmaus W. J. J. Ray M. Torres M. A. Smith A. K. (2017). Comparison of different cell type correction methods for genome-scale epigenetics studies. BMC Bioinforma. 18, 216. Epub 20170414. 10.1186/s12859-017-1611-2 PMC5391562 28410574

[B15] Levine M. E. Lu A. T. Quach A. Chen B. H. Assimes T. L. Bandinelli S. (2018). An epigenetic biomarker of aging for lifespan and healthspan. Aging (Albany NY) 10, 573–591. 10.18632/aging.101414 29676998 PMC5940111

[B16] Lo Y. H. Lin W. Y. (2022). Cardiovascular health and four epigenetic clocks. Clin. Epigenetics 14 (14), 73. Epub 20220609. 10.1186/s13148-022-01295-7 35681159 PMC9185918

[B17] Lu A. T. Quach A. Wilson J. G. Reiner A. P. Aviv A. Raj K. (2019). DNA methylation GrimAge strongly predicts lifespan and healthspan. Aging (Albany NY) 11, 303–327. 10.18632/aging.101684 30669119 PMC6366976

[B18] Maddock J. Castillo-Fernandez J. Wong A. Cooper R. Richards M. Ong K. K. (2020). DNA methylation age and physical and cognitive aging. J. Gerontol. A Biol. Sci. Med. Sci. 14 (75), 504–511. Epub 2019/10/21. 10.1093/gerona/glz246 PMC8414926 31630156

[B19] Maksimovic J. Phipson B. Oshlack A. (2016). A cross-package Bioconductor workflow for analysing methylation array data. F1000Res. 5, 1281. 10.12688/f1000research.8839.1 27347385 PMC4916993

[B20] McCrory C. Fiorito G. Hernandez B. Polidoro S. O'Halloran A. M. Hever A. (2021). GrimAge outperforms other epigenetic clocks in the prediction of age-related clinical phenotypes and all-cause mortality. J. Gerontol. A Biol. Sci. Med. Sci. 30 (76), 741–749. 10.1093/gerona/glaa286 PMC8087266 33211845

[B21] McEwen L. M. Jones M. J. Lin D. T. S. Edgar R. D. Husquin L. T. MacIsaac J. L. (2018). Systematic evaluation of DNA methylation age estimation with common preprocessing methods and the Infinium MethylationEPIC BeadChip array. Clin. Epigenetics 10, 123. 10.1186/s13148-018-0556-2 30326963 PMC6192219

[B22] Murguia-Romero M. Jimenez-Flores J. R. Sigrist-Flores S. C. Espinoza-Camacho M. A. Jimenez-Morales M. Pina E. (2013). Plasma triglyceride/HDL-cholesterol ratio, insulin resistance, and cardiometabolic risk in young adults. J. Lipid Res. 54, 2795–2799. Epub 2013/07/19. 10.1194/jlr.M040584 23863983 PMC3770092

[B23] Poulton R. Moffitt T. E. Silva P. A. (2015). The Dunedin multidisciplinary health and development study: Overview of the first 40 years, with an eye to the future. Soc. Psychiatry Psychiatr. Epidemiol. 50, 679–693. Epub 2015/04/04. 10.1007/s00127-015-1048-8 25835958 PMC4412685

[B24] Quach A. Levine M. E. Tanaka T. Lu A. T. Chen B. H. Ferrucci L. (2017). Epigenetic clock analysis of diet, exercise, education, and lifestyle factors. Aging 9, 419–446. 10.18632/aging.101168 28198702 PMC5361673

[B25] Titus A. J. Gallimore R. M. Salas L. A. Christensen B. C. (2017). Cell-type deconvolution from DNA methylation: A review of recent applications. Hum. Mol. Genet. 26, R216–R224. 10.1093/hmg/ddx275 28977446 PMC5886462

[B26] Triche T. J. Jr. Weisenberger D. J. Van Den Berg D. Laird P. W. Siegmund K. D. (2013). Low-level processing of Illumina Infinium DNA methylation BeadArrays. Nucleic Acids Res. 41, e90. 10.1093/nar/gkt090 23476028 PMC3627582

[B27] Zou H. Hastie T. (2005). Regularization and variable selection via the elastic net (vol B 67, pg 301, 2005). J. Roy. Stat. Soc. B 67, 768. 10.1111/j.1467-9868.2005.00503.x

