# Supplementary material for: Epigenetic clocks derived from western samples differentially reflect Taiwanese health outcomes
Source: Front Genet. 2023 Feb 6;14:1089819. doi: 10.3389/fgene.2023.1089819 (PMC9939687; doi:10.3389/fgene.2023.1089819)
Supplement: Supplementary file 1 [file DataSheet1.PDF]

Supplementary Materials for

Epigenetic clocks derived from Western samples differentially reflect Taiwanese health outcomes

Wan-Yu Lin [1,2,3]*

[1] Institute of Epidemiology and Preventive Medicine, College of Public Health, National Taiwan University, Taipei, Taiwan

[2] Master of Public Health Degree Program, College of Public Health, National Taiwan University, Taipei, Taiwan

[3] Department of Public Health, College of Public Health, National Taiwan University, Taipei, Taiwan

Short title: Epigenetic clocks for Taiwanese

* Corresponding author: Wan-Yu Lin, Ph.D.

Wan-Yu Lin, Ph.D.

Room 501, No. 17, Xu-Zhou Road, Taipei 100, Taiwan

Phone/Fax: +886-2-33668106; E-mail: linwy@ntu.edu.tw

https://orcid.org/0000-0002-3385-4702

13 dichotomous health outcomes	HannumEAA (s.d. = 3.63 years)				IEAA (s.d. = 3.69 years)				PhenoEAA (s.d. = 4.79 years)				GrimEAA (s.d. = 3.43 years)				DunedinPACE (s.d. = 0.1064)			
	OR	95% CI		*p*-value	OR	95% CI		*p*-value	OR	95% CI		*p*-value	OR	95% CI		*p*-value	OR	95% CI		*p*-value
**Obesity**	1.119	1.013	1.235	2.7E-02	1.124	1.018	1.241	2.0E-02	**1.220**	**1.106**	**1.347**	**7.4E-05**	**1.220**	**1.101**	**1.352**	**1.4E-04**	**1.605**	**1.450**	**1.779**	**9.6E-20**
**Adiposity**	1.214	1.069	1.380	2.9E-03	1.245	1.100	1.411	5.7E-04	1.236	1.087	1.407	1.3E-03	1.225	1.088	1.381	8.0E-04	**1.666**	**1.458**	**1.908**	**9.8E-14**
**Abdominal obesity**	1.175	1.043	1.325	8.2E-03	1.166	1.037	1.311	1.0E-02	1.218	1.078	1.377	1.6E-03	**1.294**	**1.155**	**1.451**	**8.9E-06**	**1.638**	**1.443**	**1.864**	**3.9E-14**
**Diabetes**	1.154	0.993	1.340	6.0E-02	1.092	0.940	1.268	2.5E-01	**1.326**	**1.140**	**1.544**	**2.7E-04**	**1.499**	**1.278**	**1.757**	**6.0E-07**	**1.699**	**1.461**	**1.978**	**7.0E-12**
**Hypertension**	**1.181**	**1.081**	**1.292**	**2.5E-04**	**1.194**	**1.092**	**1.307**	**1.1E-04**	**1.205**	**1.103**	**1.317**	**3.8E-05**	**1.227**	**1.115**	**1.352**	**2.8E-05**	**1.309**	**1.194**	**1.437**	**1.2E-08**
**Hypertriglyceridemia**	1.094	0.987	1.212	8.7E-02	1.082	0.977	1.198	1.3E-01	1.106	0.999	1.224	5.3E-02	**1.295**	**1.166**	**1.439**	**1.5E-06**	**1.519**	**1.369**	**1.687**	**4.5E-15**
**High LDL-C**	0.948	0.871	1.031	2.1E-01	0.948	0.872	1.031	2.1E-01	0.957	0.881	1.040	3.0E-01	0.952	0.869	1.042	2.9E-01	0.952	0.873	1.037	2.6E-01
**Low HDL-C**	1.047	0.913	1.202	5.1E-01	1.038	0.907	1.188	5.8E-01	1.255	1.091	1.446	1.6E-03	**1.400**	**1.232**	**1.593**	**2.7E-07**	**1.664**	**1.444**	**1.920**	**2.3E-12**
**High TG/HDL-C ratio**	1.138	0.996	1.301	5.7E-02	1.079	0.947	1.229	2.5E-01	**1.332**	**1.162**	**1.529**	**4.3E-05**	**1.477**	**1.303**	**1.677**	**1.2E-09**	**1.859**	**1.615**	**2.146**	**1.2E-17**
**Coronary artery disease**	1.429	1.059	1.919	1.8E-02	1.305	0.971	1.741	7.4E-02	1.359	0.993	1.859	5.5E-02	1.578	1.188	2.081	1.4E-03	1.514	1.117	2.039	6.7E-03
**Cardiovascular diseases**	1.046	0.922	1.187	4.8E-01	1.124	0.991	1.275	6.8E-02	1.012	0.892	1.148	8.5E-01	1.159	1.011	1.325	3.2E-02	1.124	0.987	1.279	7.6E-02
**Regular exercise**	0.992	0.910	1.081	8.5E-01	0.964	0.884	1.051	4.0E-01	0.864	0.793	0.942	9.3E-04	**0.825**	**0.750**	**0.907**	**6.8E-05**	**0.823**	**0.752**	**0.901**	**2.4E-05**
**Choosing walking as the regular exercise**	1.166	1.030	1.322	1.6E-02	1.113	0.977	1.269	1.1E-01	1.166	1.027	1.325	1.8E-02	1.263	1.096	1.458	1.3E-03	**1.378**	**1.203**	**1.581**	**4.4E-06**

Table S1 - (Minimally adjusted models, models adjusted only for age and sex) Odds ratio (with 95% C.I. and *p*-values) of increasing one standard deviation of epigenetic age acceleration

Significant results with $p < 0.05/(25x5) = 4.0E-4$ were highlighted in bold font.

12 continuous health outcomes	HannumEAA (s.d. = 3.63 years)				IEAA (s.d. = 3.69 years)				PhenoEAA (s.d. = 4.79 years)				GrimEAA (s.d. = 3.43 years)				DunedinPACE (s.d. = 0.1064)			
	Spearman's correlation	95% CI		*p*-value	Spearman's correlation	95% CI		*p*-value	Spearman's correlation	95% CI		*p*-value	Spearman's correlation	95% CI		*p*-value	Spearman's correlation	95% CI		*p*-value
**Body mass index**	0.060	0.021	0.099	2.9E-03	0.059	0.019	0.098	3.5E-03	**0.103**	**0.063**	**0.142**	**3.2E-07**	**0.119**	**0.080**	**0.158**	**3.0E-09**	**0.251**	**0.213**	**0.288**	**6.8E-37**
**Body fat percentage**	0.068	0.028	0.108	9.2E-04	0.046	0.006	0.086	2.4E-02	**0.092**	**0.052**	**0.132**	**6.5E-06**	**0.111**	**0.071**	**0.150**	**5.9E-08**	**0.235**	**0.196**	**0.273**	**2.5E-31**
**Waist circumference**	0.059	0.020	0.099	3.1E-03	0.051	0.011	0.090	1.1E-02	**0.100**	**0.060**	**0.139**	**7.0E-07**	**0.124**	**0.085**	**0.163**	**6.7E-10**	**0.228**	**0.190**	**0.265**	**1.7E-30**
**Fasting glucose**	0.031	-0.008	0.071	1.2E-01	-0.016	-0.055	0.024	4.3E-01	0.071	0.032	0.110	4.1E-04	**0.076**	**0.037**	**0.115**	**1.5E-04**	0.053	0.014	0.092	8.4E-03
**HbA1c**	0.015	-0.025	0.054	4.7E-01	0.000	-0.039	0.040	9.9E-01	0.054	0.015	0.093	7.0E-03	**0.080**	**0.040**	**0.119**	**7.4E-05**	**0.116**	**0.077**	**0.155**	**6.6E-09**
**Diastolic blood pressure**	0.067	0.028	0.107	8.2E-04	0.069	0.029	0.108	6.1E-04	**0.078**	**0.038**	**0.117**	**1.1E-04**	0.041	0.002	0.081	4.0E-02	**0.107**	**0.068**	**0.146**	**9.0E-08**
**Systolic blood pressure**	0.069	0.029	0.108	6.3E-04	0.034	-0.005	0.074	8.7E-02	**0.073**	**0.034**	**0.113**	**2.6E-04**	0.064	0.024	0.103	1.5E-03	**0.121**	**0.081**	**0.159**	**1.8E-09**
**Triglyceride**	0.001	-0.038	0.040	9.6E-01	0.005	-0.034	0.044	8.0E-01	0.063	0.023	0.102	1.8E-03	0.071	0.031	0.110	4.2E-04	**0.176**	**0.137**	**0.214**	**1.3E-18**
**LDL-C**	-0.015	-0.054	0.025	4.6E-01	-0.015	-0.055	0.024	4.4E-01	-0.033	-0.072	0.006	1.0E-01	-0.021	-0.061	0.018	2.9E-01	-0.027	-0.066	0.013	1.9E-01
**HDL-C**	-0.058	-0.097	-0.019	4.0E-03	-0.023	-0.063	0.016	2.5E-01	**-0.078**	**-0.117**	**-0.039**	**9.9E-05**	**-0.135**	**-0.173**	**-0.095**	**1.9E-11**	**-0.239**	**-0.276**	**-0.201**	**2.0E-33**
**TG/HDL-C ratio**	0.010	-0.030	0.049	6.4E-01	0.001	-0.038	0.041	9.5E-01	**0.078**	**0.039**	**0.117**	**1.1E-04**	0.068	0.029	0.107	7.0E-04	**0.205**	**0.166**	**0.242**	**8.7E-25**
**Forced vital capacity**	-0.035	-0.085	0.014	1.6E-01	-0.008	-0.057	0.041	7.5E-01	-0.036	-0.085	0.014	1.6E-01	-0.041	-0.091	0.008	1.0E-01	-0.052	-0.102	-0.003	3.8E-02

Table S2 - (Minimally adjusted models, models adjusted only for age and sex) Spearman's rank correlation coefficients (with 95% C.I. and *p*-values) between epigenetic age acceleration and continuous health outcomes

Significant results with $p < 0.05/(25x5) = 4.0E-4$ were highlighted in bold font.
